# How Universal Health Coverage can curb the escalating antimicrobial resistance in Pakistan: a call to action for the country's healthcare system

**DOI:** 10.1186/s41182-022-00478-5

**Published:** 2022-11-14

**Authors:** Laiba Imran, Syeda Tayyaba Rehan, Ka Yiu Lee

**Affiliations:** 1 Dow University of Health Sciences, Karachi, 74200 Pakistan; 2 Department of Health Sciences, Swedish Winter Sports Research Centre, Mid Sweden University, Östersund, Sweden

## Abstract

Antimicrobial resistance (AMR) has emerged as a major threat to the global healthcare economy during Coronavirus disease 2019 (COVID-19), especially in developing countries like Pakistan where the healthcare facilities are already substandard. To combat AMR, the World Health Organization (WHO) has taken several initiatives including the establishment of a sustainable Universal Health Coverage (UHC) system. The implementation of UHC could eliminate various factors that contribute to a high AMR rate including self-medication. Our commentary explores in depth the current UHC system in Pakistan and how UHC could be the answer to Pakistan’s AMR crisis.

For decades developing countries have been plagued by increasing rates of antimicrobial resistance (AMR), a consequence of gross overconsumption of antibiotics [1]. The increasing incidence of AMR poses a major threat to the healthcare economy worldwide and has been declared a public health emergency by the World Health Organization (WHO) [2]. Research shows that several middle or low-income countries including India, Bangladesh, Afghanistan, and Pakistan have some of the highest rates of AMR across the globe [3] which can be attributed to the poor and inadequate medical facilities available in these countries [4]. Other factors that increase the potential for AMR transmission include the use of antimicrobials in agriculture and animal poultry, making AMR a threat to animal and ultimately human health [5].

Pakistan’s healthcare system (HCS) is among those which are severely threatened by AMR as evidence shows that most bacteria have developed strong resistance to frequently used antibiotics in the country [2], rendering the antibiotic treatment ineffective for patients. A survey from the past decade showed the prevalence of methicillin-resistant *Staphylococcus aureus* (MRSA) to be 49% and the resistant genes of *Shigella, Klebsiella, Enterococcus, Acinetobacter*, and *E. coli* were found to be 79%, 33%, 45%, 1.6% and 31% prevalent in the country, respectively [2].

The situation was made worse worldwide when the COVID-19 pandemic hit the globe, as the consumption of antibiotics increased by 23.3% during the pandemic [6] and an exacerbation was seen in the rate of AMR [7]. This rise in AMR is not only concerning for the present deteriorating health conditions in the country, but could also be an alarming sign of innumerable untreated future maladies.

The high incidence of AMR in Pakistan can mostly be attributed to illiteracy that is prevalent throughout the country. Lack of awareness means that patients consume antibiotics as they see fit, their only concern being fast relief without any regard for the adverse effects of the antibiotics [8]. Studies disclosed that between 2000 and 2015, a 65% escalation was observed in antibiotic consumption in the country [6]. Another factor that contributes to self-medication is the easy access to most antibiotics as over-the-counter medicines without doctors' prescription [9]. Moreover, the majority of the population struggles financially and hence cannot afford frequent doctor visits, especially for complaints like cough or headache, further contributing to the problem of self-medication. It is estimated that more than 500,000 people die annually in the country due to issues like self-medication and wrong prescription [6]. Distrust of doctors or the healthcare system, in general, is also a common sentiment among the public where people believe that doctors recommend more medication than necessary. Due to such misconceptions, people try to steer clear of proper doctor visits unless it becomes necessary or their condition becomes too critical [10]. Those who do visit a doctor and get prescribed antibiotics, usually do not complete the antibiotic course and stop taking medication as soon as they feel better [11], further worsening the AMR situation in Pakistan.

Since AMR is a major burden on the global healthcare economy, WHO has launched several initiatives to combat its increase. In order to combat AMR, it is essential that an integrated One Health approach is taken that incorporates human health, animal health and the environment when considering the management of AMR [12]. One of the key initiatives focusing on human health includes the implementation of Universal Health Coverage (UHC) as a means of making healthcare affordable for all populations, especially the lower class [13]. UHC is defined as obtaining access to important healthcare services such as disease prevention, treatment, and health improvement [14] and is of utmost importance, especially in countries like Pakistan, where most of the population cannot afford the burden of quality healthcare services as Pakistan does not have a national health insurance system and 78% of the population pays health care expenses themselves [15]. UHC, if implemented throughout the country, can prove to be crucial in solving Pakistan’s AMR crisis. If healthcare does not pose a major financial burden, people will start leaning towards doctor visits instead of just self-medicating. This will also provide doctors with the opportunity of counseling their patients about antibiotic resistance and they can emphasize the importance of completing the antibiotic course which can help reduce the AMR rate significantly. Due to a lack of awareness, most people also self-medicate using antibiotics even when they have a viral infection. UHC could eliminate this obstacle by ensuring individuals access to healthcare providers who can prescribe the right regimen for their ailment.

In recent years, Pakistan has taken several steps to establish UHC within the country. A health card has been introduced in two provinces, Khyber Pakhtunkhwa and Punjab, which has made healthcare affordable for even the underprivileged [16]. The health card allows each family to receive treatments worth 1 million PKR annually in both government and private hospitals and covers all costs related to hospitalization for chronic diseases, allowing individuals to undergo expensive treatments that would have otherwise left them bankrupt [16]. During the first seven months of 2021, 250,439 patients were treated free of cost in government and private hospitals just in the city of Peshawar in KPK [17], shedding light on the importance of the health card in the citizens’ lives. However, since the country has been facing political instability lately, the fate of UHC also hangs in balance where some hospitals have postponed treatments for patients using health cards [18]. Moreover, UHC has not been implemented in other provinces, which has only increased the healthcare disparity that was already evident within the country.

## Call for action

We suggest the following actions need to be considered for establishing UHC in the country and to uproot the AMR crisis:

- Health cards should be available for citizens throughout the country, not just in specific provinces.
- The healthcare sector should collaborate with the media to spread maximum awareness regarding antibiotic resistance and how it can be prevented.
- All healthcare workers should be properly educated on AMR so they do not prescribe antibiotics unless absolutely necessary as trends show that the prescribing of medications by inadequately trained pharmacists and unlicensed practitioners only further worsens the AMR situation in the country [19].
- Free health clinics should be organized frequently in underdeveloped areas with detailed sessions on antibiotic resistance and how it is fuelled by self-medication.
- Pharmacies should not be authorized to sell antibiotics without a doctor’s prescription and should be mandated by the law to keep a track of all antimicrobials sold in order to accurately evaluate antibiotic consumption among the population so the AMR problem can be tackled accordingly.

## Data Availability

Not applicable.
